# Anatomical, Physiological and Transcriptomic Insights into Salt Tolerance in Two Peanut Lines with Different Oil Contents

**DOI:** 10.3390/plants15081193

**Published:** 2026-04-13

**Authors:** Xiuhua Yao, Chunmei Zhao, Yan Li, Min Cao, Yue Liu

**Affiliations:** 1 College of Horticulture, Qingdao Agricultural University, Qingdao 266109, China; 2 College of Life Science, Qingdao Agricultural University, Qingdao 266109, China

**Keywords:** peanut, salt tolerance, structure, physiological response, transcriptome

## Abstract

Soil salinization is a significant global challenge that severely impacts agricultural productivity, particularly through its negative effects on crop growth and yield. Peanuts (*Arachis hypogaea* L.) are an important oil crop. One of the major goals in peanut breeding programs is to develop varieties with both high oil content and salt tolerance. Previously, we obtained a peanut line (HO) with high oil content through mutagenesis, which showed higher salt tolerance than its parental line (HY20). In this study, we employed multiple approaches including anatomical, physiological, and transcriptomic analyses to elucidate salt tolerance mechanisms of the HO peanut line. Under salt stress, the HO line exhibited better-developed vascular structures, with increased root vessel diameter and higher crystal idioblast density in leaves compared to HY20. HO also showed enhanced antioxidant enzyme activities, with POD and SOD activities higher than HY20. Photosynthetic efficiency was substantially improved in HO, with Fv/Fm decreasing under severe salt stress. Additionally, HO maintained a lower Na^+^/K^+^ ratio and higher linolenic acid content under salt stress. Transcriptomic analysis revealed up-regulated lignin biosynthesis genes in HO. This study established potential connections between salt stress tolerance and oil biosynthesis in peanuts, providing insights that could be leveraged for the development of high-yield and salt-resistant varieties.

## 1. Introduction

Soil salinization is a significant constraint on agricultural development worldwide. It impairs crop growth and development, leading to reduced yields and quality. Increased salinity can have detrimental effects on plant physiology, including oxidative stress and ionic toxicity [1]. Oxidative stress is characterized by an overproduction of reactive oxygen species (ROS), such as hydrogen peroxide (H_2_O_2_) and superoxide anions (O_2_^•−^) [2]. These ROS can cause significant damage to cellular components, including the peroxidation of membrane lipids, which impairs membrane integrity and function [3]. Under abiotic stress, antioxidant enzymes such as superoxide dismutase (SOD), peroxidase (POD), and catalase (CAT) can effectively remove ROS and alleviate oxidative stress, helping to maintain the growth of plants [4]. In addition to oxidative stress, salt stress can also lead to ionic imbalances within the plant. An increase in Na^+^ content is often accompanied by a decrease in the levels of potassium (K^+^) and calcium (Ca^2+^), which can result in Na^+^ toxicity [5]. This toxicity can disrupt cellular processes and interfere with the plant’s ability to maintain osmotic balance and nutrient uptake. One environmentally friendly approach to mitigating soil salinization is the use of plants that can tolerate high salinity levels. These plants have the ability to draw excess salt from the soil, which also enhances the soil’s organic matter and nutrient content. Consequently, identifying and cultivating salt-tolerant plants has emerged as an effective strategy for developing and utilizing saline lands.

Peanut (*Arachis hypogaea* L.), containing 36–54% oil, 16–36% protein, and 10–20% carbohydrates, is a globally important oil crops with strategic importance in the development of agriculture and the national economy [6,7]. It is widely cultivated in Asia, Africa, and America [8]. Nearly half of the peanuts produced in China are used for oil extraction, highlighting the crop’s economic importance. Peanuts are commonly planted in arid and semiarid hilly regions in China due to their higher tolerance to barren conditions [9]. Despite their moderate salt tolerance, peanuts are not commonly cultivated in saline-alkali lands within China due to the limited availability of high-yielding, high-oil-content peanut varieties that also possess the necessary salt tolerance [10,11]. Therefore, innovation in peanut germplasm to improve yield, oil content, and salt tolerance is a significant task in peanut research.

To achieve this goal, there has been extensive research on understanding the salt tolerance mechanisms and genetic basis of peanuts. At the physiological level, salt-tolerant peanut genotypes exhibit enhanced capacity for maintaining Na^+^/K^+^ homeostasis, greater antioxidative enzyme activities (SOD, POD, CAT), and increased accumulation of osmoprotectants such as proline and sugars [12]. The publication of the whole-genome sequences of peanut has helped elucidate various biological processes in peanut through omics methods [13,14,15,16,17,18]. In recent years, an increasing number of peanut salt-stress response genes have been identified through transcriptome analysis, including aquaporin isoform AhTIP3;1 [19], ABA biosynthesis key enzymes AAO2 and AAO3 [20], Glutamyl-tRNA reductase1 (HEMA1) and ferrochelatase1 (FC1) [21], bZIP [22], NAC [23], AP2/ERF [24], HSF [25], WRKY [26] and bHLH [27] transcription factors. Genes involved in fatty acid biosynthesis and metabolism were related to salt tolerance [28,29]. Through transcriptome analysis, some researchers have found some genes related to oil biosynthesis [30,31].

Despite these advances, most studies have focused on either salt tolerance or oil biosynthesis in isolation. The mechanistic link between oil accumulation and salt tolerance remains poorly understood, hindering the development of superior varieties that can offer high yields and oil content while withstanding the challenges of saline soils. In 2020, a practical and reliable method was established for creating new peanut varieties with high oil content using in vitro mutagenesis and directional osmotic pressure (OP)-based selection [11]. Using this method, a genetically stable high-oil peanut line with oil content >55% was developed through pingyangmycin (PYM) mutagenesis, with hydroxyproline (HYP) as a selection factor. Notably, the high-oil-content line (HO, 58% oil content) exhibited better growth and yield performance when cultivated in saline soils compared to its parental line Huayu 20 (HY20, 52% oil content), suggesting a potential connection between oil content and salt tolerance. This pair of materials can eliminate the interference of unrelated genetic background, making them ideal models to explore the link between high oil content and salt tolerance in peanut.

In this study, we used two contrasting peanut genotypes: HO derived from the parental cultivar HY20, and its wild-type parent HY20, to systematically investigate the relationship between high oil content and salt tolerance in peanut. We further dissected the underlying salt tolerance mechanisms of the two lines through integrated anatomical, physiological, and transcriptomic analyses. Elucidating the mechanistic link between oil accumulation and salt tolerance in peanut will provide a solid theoretical basis for breeding high-oil, salt-tolerant peanut varieties adapted to saline-alkali soils.

## 2. Results

### 2.1. Salt Tolerance in High-Oil Peanuts and Its Mutagenic Parent of Peanuts

**Figure 1:**
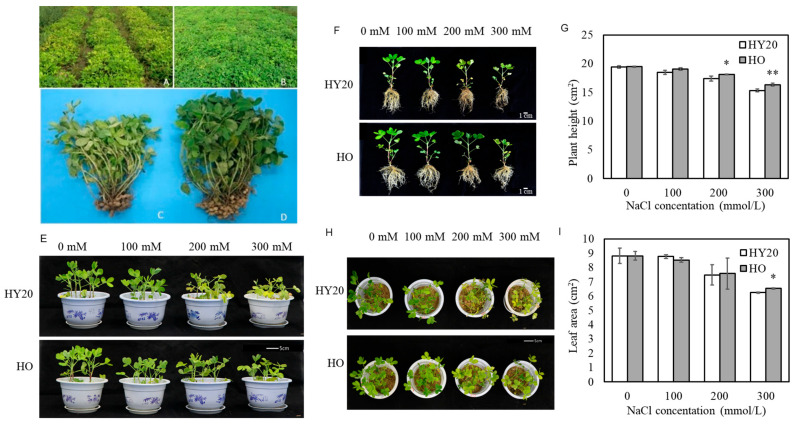
Phenotypic responses of HY20 and HO peanut lines to salt stress. (**A**,**B**) HY20 (**A**) and HO (**B**) plants grown in saline-alkali soil. (**C**,**D**) Close-up views of HY20 (**C**) and HO (**D**) grown in saline-alkali soil. (**E**,**H**) Seedlings of HY20 and HO in an artificial climate chamber under control conditions: side view (**E**) and top view (**H**). (**F**) Plants of HY20 and HO under salt treatment. (**G**) Plant height. (**I**) Leaf area. Data are mean ± SE (*n* = 3). Student’s *t*-test was used for pairwise comparisons; *p* < 0.05 (*), *p* < 0.01 (**). HO = high-oil line; HY20 = parental line.

Previously, we obtained a stable, high-oil content peanut line (HO), which was selected through hydroxyproline screening following mutagenesis with Pingyangmycin [11]. We re-measured the oil content of HO and its parental line (HY20) and found that HO seeds contain an average oil content of 58%, surpassing the 52% oil content in HY20 seeds. When planted in saline-alkali soils, the HO line exhibits better growth and yield compared to HY20 (Figure 1A–D). Additionally, HO seedlings exhibited enhanced growth under salt stress conditions in an artificial climate chamber compared to HY20 (Figure 1E). After NaCl treatment, Upon the HO line exhibited higher plant height than the HY20 line (Figure 1F), with statistically significant differences observed at 200 mmol/L and 300 mmol/L NaCl (Figure 1G). The wilting and yellowing of leaves occurred more rapidly in HY20 than in the HO line after NaCl treatment (Figure 1H), with a more pronounced difference in growth at higher NaCl concentrations. Specifically, the leaf area of the HO line was reduced by 25.95% in response to treatment, whereas the HY20 line experienced a 29.19% decrease (Figure 1I). These results indicate that the HO line exhibits better growth performance under salt stress than its parental line HY20.

### 2.2. Anatomy Changes in Response to Salt Stress in HO and HY20

**Figure 2:**
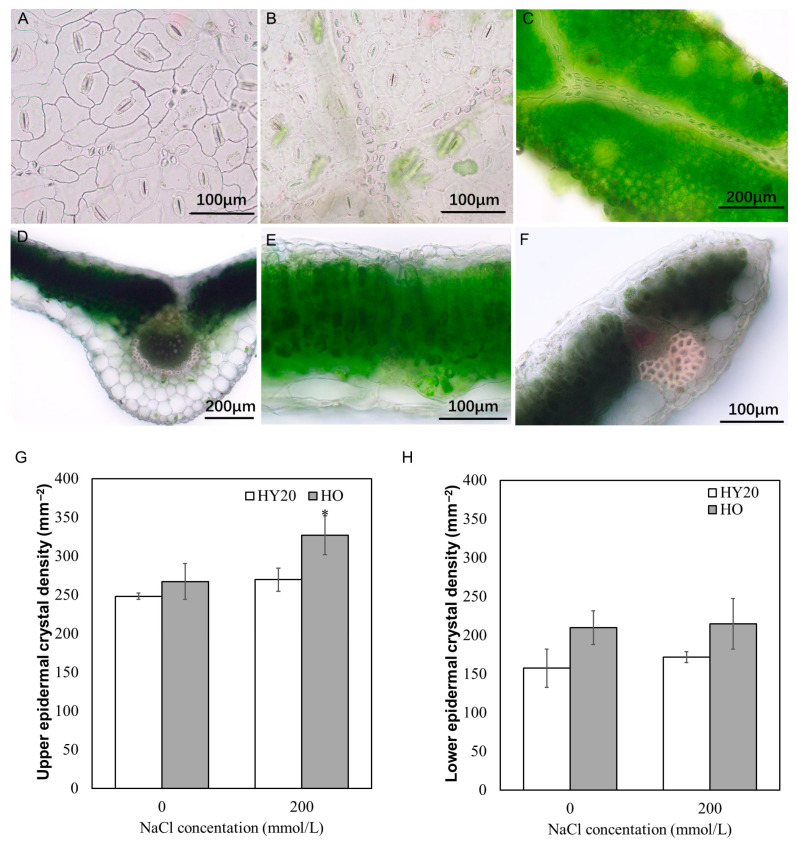
Salt tolerant structure of peanut leaves. (**A**) Lower epidermis. (**B**) Upper epidermis. (**C**) Upper leaf surface showing crystal idioblasts. (**D**) Cross-section showing main veins and water storage cells. (**E**) Cross-section showing mesophyll cells and water storage cells. (**F**) Cross-section showing lateral veins and water storage cells. (**G**) Crystal density in upper epidermis. (**H**) Crystal density in lower epidermis. Data are mean ± SE (*n* = 3). Student’s *t*-test was used for pairwise comparisons; *p* < 0.05 (*). HO = high-oil line; HY20 = parental line.

**Figure 3:**
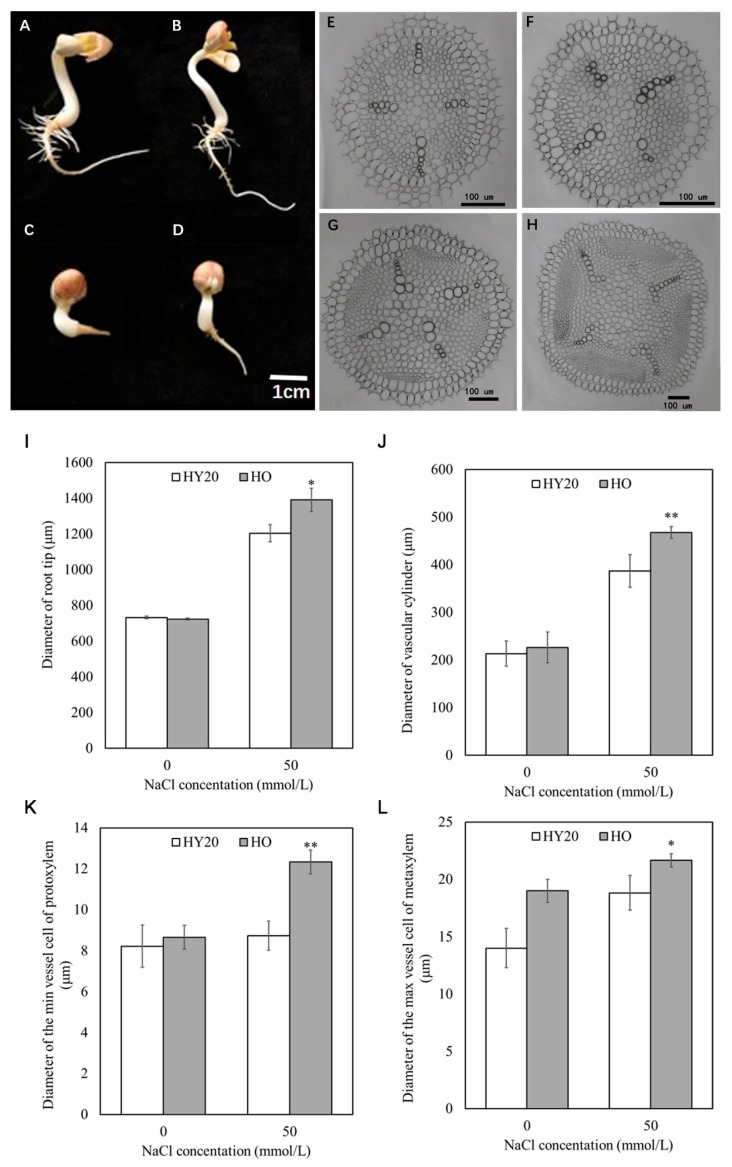
Changes of salt tolerance structure of peanut rootwith and without salt stress. (**A**,**B**) Seedling roots under control conditions: HY20 (**A**) and HO (**B**). (**C**,**D**) Seedling roots treated with 50 mmol/L NaCl: HY20 (**C**) and HO (**D**). (**E**,**F**) Cross-sections of root tips (1 cm from tip) under control conditions: HY20 (**E**) and HO (**F**). (**G**,**H**) Cross-sections under mmol/L NaCl: HY20 (**G**) and HO (**H**). (**I**) Root diameter. (**J**) Vascular cylinder diameter. (**K**) Protoxylem vessel diameter. (**L**) Metaxylem vessel diameter. Data are mean ± SE (*n* = 3). Student’s *t*-test was used for pairwise comparisons; *p* < 0.05 (*), *p* < 0.01 (**). HO = high-oil line; HY20 = parental line.

Peanuts exhibit moderate salt tolerance and have a well-organized plant structure that supports their ability to withstand barren and saline conditions. Peanut plants possess a dense covering of epidermal hairs, a thick cutin membrane on the leaf epidermis, and stomata predominantly distributed on the lower epidermis. The mesophyll contains large water storage tissues, well-developed palisade and mechanical tissues, and mesophyll cells rich in chloroplasts. Abundant crystal idioblasts (containing calcium oxalate) are present in the epidermis and mesophyll, particularly rich in the upper epidermis (Figure 2A–F). The roots of peanut are tetrarch. The cell walls of the epidermis in their primary roots are thickened and composed of multiple layers of cells, which are lignified to prevent the entry of harmful salt ions. The vascular bundle occupies a large area of the cross-section of roots, and the pericycle is well-developed. These features suggest that the peanut roots have a strong ability to protect, retain, and transport water (Figure 3).

Then, we analyzed the impact of NaCl treatment on the structure of roots and leaves. It was found that the number of crystal idioblasts in the upper and lower epidermis of both HO and HY20 leaves was increased after treatment with 200 mmol/L NaCl for 10 days (Figure 2G,H). Notably, the number of crystal idioblasts in the upper epidermis of HO leaves increased more significantly compared with HY20. This suggests that crystal idioblast is an adaptive structural feature of peanut for salt tolerance.

After treating peanut HY20 and HO seedlings with 50 mmol/L NaCl solution and culturing for 8 days, it was found that the growth of peanut HY20 and HO roots was inhibited under salt stress, and the number of lateral roots decreased. However, the length of root of the HO line was significantly better than that of HY20 (Figure 3A–D). The cross-section of peanut HY20 and HO roots at a distance of 1 cm from the root tip showed that salt stress promoted the development of the xylem of roots and accelerated the differentiation of vessels. The differentiation of vessels in HO was significantly earlier than in HY20 (Figure 3E,F). We found that after salt stress treatment, the diameter of the root’s cross-section and the diameter of the vascular cylinder of both peanut HY20 and HO increased (Figure 3I,J). Moreover, HO not only significantly increased the diameter of the root’s cross-section and the diameter of the vascular column compared to HY20, but also significantly increased the diameter of the protoxylem and metaxylem (Figure 3K,L).

### 2.3. Biochemical and Phsyiological Changes in HO and HY20 in Responses to Salt Stress

**Figure 4:**
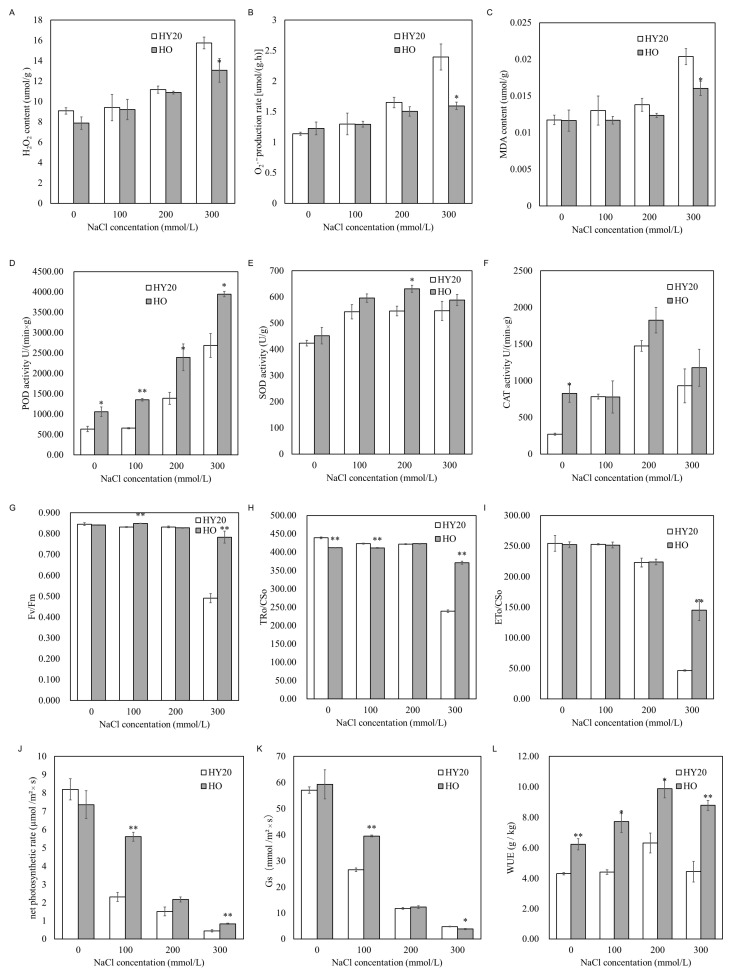
Physiological responses of HO and HY20 peanut lines to salt stress. (**A**) Hydrogen peroxide (H_2_O_2_) content in leaves. (**B**) Superoxide anion (O_2_•^−^) content in leaves. (**C**) Malondialdehyde (MDA) content in leaves. (**D**) Peroxidase (POD) activity in leaves. (**E**) Superoxide dismutase (SOD) activity in leaves. (**F**) Catalase (CAT) activity in leaves. (**G**) Maximum photochemical efficiency of PSII (Fv/Fm). (**H**) Trapped energy flux per cross-section (TRo/CSo). (**I**) Electron transport flux per cross-section (ETo/CSo). (**J**) Net photosynthetic rate. (**K**) Stomatal conductance (Gs). (**L**) Water use efficiency (WUE). Data are mean ± SE (*n* = 3). Student’s *t*-test was used for pairwise comparisons; *p* < 0.05 (*), *p* < 0.01 (**). HO = high-oil line; HY20 = parental line.

We analyzed the physiological changes in the leaves of HO and HY20 under salt stress. As the salt treatment concentration increased, the content of H_2_O_2_ and O_2_^•−^ in the leaves of both HO and HY20 increased. However, the levels of H_2_O_2_ and O_2_^•−^ in HO leaves were consistently lower than in HY20. Notably, after treatment with 300 mmol/L NaCl, the rate of O_2_^•−^ production in HO leaves was significantly lower, reaching only 66.6% of that in HY20 (Figure 4A,B). Upon treatment with 300 mmol/L, HO accumulated less Malondialdehyde (MDA) than HY20 leaves (Figure 4C). These suggest that the HO line can better resist oxidative stress caused by salt treatment.

During salt stress, the activities of the antioxidant enzymes SOD, CAT, and POD generally increased in both the HO and HY20 peanut lines. However, the increases were more pronounced in HO compared to HY20, particularly for POD activity (Figure 4D–F). Prior to salt treatment, the activities of POD and CAT in the leaves of the HO line were already higher than those in the HY20 line. Under salt stress, the POD and SOD activities in HO leaves were higher than in HY20. These findings suggest that the HO line has a heightened capacity for antioxidant activity, allowing it to scavenge reactive oxygen species more effectively than HY20. This enhanced ability to scavenge reactive oxygen species (ROS) may be an important factor contributing to the HO line’s improved salt tolerance compared to HY20.

The HO line exhibited higher photosynthetic efficiency than HY20, as evidenced by measurements of chlorophyll fluorescence parameters of Photosystem II (PS II) (Figure 4G–I). As salt concentration increases, there is a general decline in the key chlorophyll fluorescence parameters Fv/Fm (the maximum photochemical efficiency), TRo/CSo (trapped energy flux), and ETo/CSo (electron transport flux) in both peanut lines. After 11 days of treatment with 300 mmol/L NaCl, the Fv/Fm value of HY20 decreased by 41.86% compared to the control, while the Fv/Fm value of HO decreased by only 6.9% compared to the control. Similarly, the TRo/CSo value of HY20 decreased by 45.43% compared to the control, whereas the TRo/CSo value of HO decreased by only 9.6% compared to the control. The ETo/CSo in HO leaves, representing the energy used by PS II for electron transfer, was found to be 3.12 times higher in HO leaves compared to HY20. These findings suggest that the HO line can minimize damage to PS II and maintain electron transfer activity to resist salt stress more effectively than HY20.

The net photosynthetic rate and stomatal conductance (Gs) of peanut seedlings decreased after salt stress. Under 100 and 300 mmol/L NaCl treatment, the net photosynthetic rate and stomatal conductance of HO were higher than those of HY20 (Figure 4J,K). Additionally, the water use efficiency (WUE) of HO line remained superior to that of the HY20 line across various salt concentrations (Figure 4L). This suggests that the HO line has a better ability to mitigate the negative impacts of salt stress, potentially by reducing water loss and maintaining more efficient photosynthetic processes.

**Figure 5:**
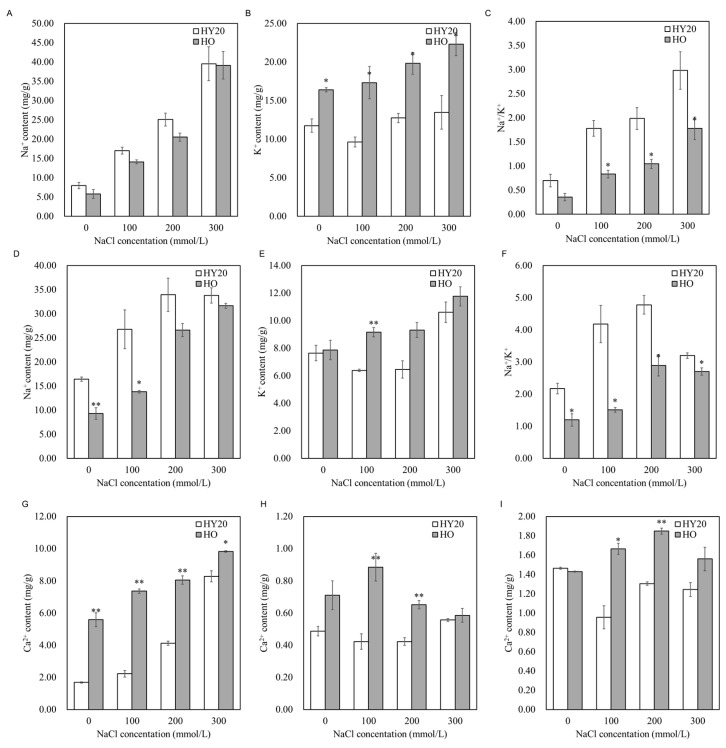
Ion homeostasis in HO and HY20 peanut lines under salt stress. (**A**) Na^+^ content in leaves. (**B**) K^+^ content in leaves. (**C**) Na^+^/K^+^ ratio in leaves. (**D**) Na^+^ content in roots. (**E**) K^+^ content in roots. (**F**) Na^+^/K^+^ ratio in roots. (**G**) Ca^2+^ content in leaves. (**H**) Ca^2+^ content in roots. (**I**) Ca^2+^ content in stems. Data are mean ± SE (*n* = 3). Student’s *t*-test was used for pairwise comparisons; *p* < 0.05 (*), *p* < 0.01 (**). HO = high-oil line; HY20 = parental line.

We examined the levels of sodium (Na^+^) and potassium (K^+^) in the roots and leaves of HO and HY20. Our findings indicate that the Na^+^/K^+^ ratio in the roots, and leaves of HO was consistently lower than in HY20, regardless of whether they were subjected to salt stress. Under various salt concentrations, HO accumulated lower Na^+^ in leaves and roots compared to HY20, while maintaining higher K^+^ levels. The Na^+^/K^+^ ratio in the leaves of HO was lower than in HY20 (Figure 5A–C). In contrast, the calcium (Ca^2+^) content in the leaves of HO was higher than in HY20 (Figure 5G). With increasing salt concentration, the Ca^2+^ content in various tissues of HO seedlings was consistently higher than those in HY20 (Figure 5G–I). The Ca^2+^ content in leaves gradually increased with increasing salt concentration, while in roots, it showed a trend of initially increasing and then decreasing (Figure 5G,H). Under 100 and 200 mmol/L salt treatment, the Ca^2+^ content in the roots and stems of HO was significantly higher than in HY20 (Figure 5H,I). These findings suggest that HO can maintain relatively low levels of Na^+^/K^+^ and relatively high levels of Ca^2+^ under salt stress.

**Figure 6:**
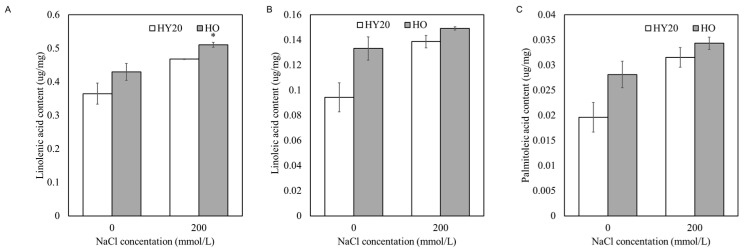
Unsaturated fatty acid content in leaves of HO and HY20 peanut lines under salt stress. (**A**) Linolenic acid. (**B**) Linoleic acid. (**C**) Palmitoleic acid. Student’s *t*-test was used for pairwise comparisons; *p* < 0.05 (*). HO = high-oil line; HY20 = parental line.

Unsaturated fatty acids, such as palmitic, linoleic, and linolenic acids, play a critical role in the composition of cell membrane phospholipids, which are essential for maintaining membrane fluidity and function. After treatment with 200 mmol/L salt, the content of linolenic acid, the highest among these unsaturated fatty acids, increased in both HO and HY20 (Figure 6A–C). Notably, the content of linolenic acid in HO peanut leaves was significantly higher than in HY20 (Figure 6A). Our results suggest that HO may contribute to reduce salt stress damage by adjusting the composition of membrane lipids. These physiological and biochemical differences between HO and HY20 prompted us to investigate the underlying transcriptional regulation.

### 2.4. Identification of DEGs During Salt Treatment in HO and HY20

To explore the molecular basis underlying the superior structural, physiological, and biochemical responses of HO under salt stress, we conducted RNA-seq analysis to compare genome-wide gene expression between the two peanut lines. One-month-old peanut leaves from HO and HY20, treated with NaCl and untreated controls, were harvested for RNA-seq analysis, with three independent biological replicates for each sample (Supplementary Table S1). We utilized stringent criteria to identify differentially expressed genes (DEGs), setting a threshold of log_2_(fold change) ≥ |1| and a false discovery rate (FDR) ≤ 0.05. Under salt stress, a total of 2876 genes were differentially expressed in the two genotypes, comprising 1149 up-regulated and 1139 down-regulated genes in HY20, and 432 up-regulated and 458 down-regulated genes in HO (Supplementary Figure S1A,B; Supplementary Data S1 and S2). The changes in gene expression in HO under salt treatment are much smaller than those in HY20. Among these, only 164 and 136 common genes were both up- and down-regulated during salt stress in HY20 and HO, respectively. This result shows the genetic complexity and line-specific responses to salt.

To gain insights into the biological processes associated with the genes regulated to salt stress, we performed Gene Ontology (GO) enrichment analysis on the combined set of differentially expressed genes identified across both genotypes (HY20 and HO) (Supplementary Figure S1C,D, Supplementary Data S3). Significantly enriched GO terms among the up-regulated genes under salt stress included “hyperosmotic salinity response” (GO:0042538, FDR = 1.40 × 10^−6^), which is directly linked to the response to high salt conditions. The following GO terms were also significantly enriched: “Jasmonic acid-mediated signaling pathway” [GO:0009867, FDR = 5.8 × 10^−35^], “Salicylic acid-mediated signaling pathway” [GO:0009862, FDR = 9.70 × 10^−23^], “Jasmonic acid biosynthetic process” [GO:0009695, FDR = 2.00 × 10^−11^], “Ethylene-activated signaling pathway” [GO:0009873, FDR = 0.00095], “Regulation of hydrogen peroxide metabolic process” [GO:0010310, FDR = 1.00 × 10^−17^], and “Leaf senescence” [GO:0010150, FDR = 0.00098]. These terms are associated with hormone signaling pathways and responses to abiotic stress. Among down-regulated genes under salt stress, the following GO terms were significantly enriched: “Regulation of meristem growth” [GO: 0010075, FDR = 3.3 × 10^−21^], “Response to red light” [GO:0010114, FDR = 3.00 × 10^−5^], “Regulation of photosynthesis, light reaction” [GO: 0042548, FDR = 0.0022], “Acetyl-CoA metabolic process” [GO:0006084, FDR = 0.0017], “Very long-chain fatty acid metabolic process” [GO:0000038, FDR = 1.40 × 10^−6^], and “Omega-3 fatty acid desaturase activity” [GO: 0042389, FDR = 0.00017]. These suggest that salt stress impacts the expression of genes involved in lipid metabolism and other stress-related processes, indicating a complex regulatory network that adjusts to saline conditions.

### 2.5. Transcriptome Analysis for Difference Between HO and HY20

**Figure 7:**
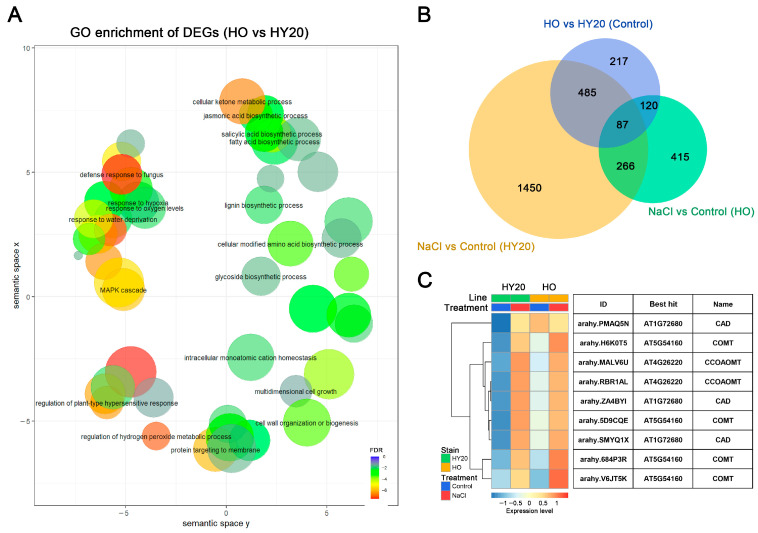
Transcriptome analysis of HY20 and HO during salt stress. (**A**) GO enrichment analysis of DEGs between HY20 and HO. Enriched GO terms are shown in two-dimensional space derived from semantic similarities. Bubble color indicates log_2_FDR; bubble size indicates term frequency in the GOA database. (**B**) Venn diagram showing overlap of DEGs between pairwise comparisons. (**C**) Expression levels of lignin biosynthesis genes in HY20 and HO under control and salt stress conditions.

To understand the genetic factors influencing oil content variation in peanuts, we identified DEGs between two peanut lines with distinct oil content levels, HO and HY20, under normal (control) conditions. We identified 525 genes with higher expression and 384 genes with lower expression in HO compared to HY20 under control conditions (Supplementary Data S4). Through GO enrichment analysis, we explored the differentially expressed biological processes between HO and HY20. The results showed that HO peanuts had differences in regulating signal transduction mechanisms, cell wall organization, defense response, and lignin-related processes (Figure 7A, Supplementary Data S5).

Among the highly expressed genes in the HO line, GO terms related to response to abiotic stress were enriched, including “Jasmonic acid-mediated signaling pathway” [GO:0009867, FDR = 6.30 × 10^−10^], “Regulation of hydrogen peroxide metabolic process” [GO:0010310, FDR = 1.00 × 10^−9^], “Regulation of salicylic acid biosynthetic process” [GO:0080142, FDR = 9.70 × 10^−6^], and “Response to water deprivation” [GO:0009414, FDR = 0.011] (Supplementary Data S5). These enriched GO terms were similar to those found in up-regulated genes treated with salt stress. Among the lowly expressed genes in the HO line, the enriched GO terms were also similar to those in down-regulated genes treated with salt stress (Supplementary Figure S2). For example, “Glucuronoxylan metabolic process” [GO:0010413, FDR = 3.40 × 10^−7^], “Xylan biosynthetic process” [GO:0045492, FDR = 3.40 × 10^−7^], “Lignin catabolic process” [GO:0046274, FDR = 8.40 × 10^−5^], and “Linoleate 13S-lipoxygenase activity” [GO:0016165, FDR = 0.0019] were significantly enriched (Supplementary Data S5). These findings suggest that the HO peanut line may have a distinct genetic profile that influences its response to stress.

To further explore the relationship between the HO transcriptome and salt tolerance, we conducted a comparative analysis of the DEGs between HO and HY20, and those genes whose expression is influenced by salt stress. We found that 76% of the DEGs between HO and HY20 had significantly changed in expression after salt treatment (Figure 7B). There were more overlaps (327 genes) between highly expressed genes in the HO line and up-regulated genes than down-regulated genes under salt stress, and more overlaps (241 genes) between lowly expressed genes in the HO line and down-regulated genes than up-regulated genes under salt stress (Supplementary Figure S3). These results suggest that a subset of DEGs between HO and HY20 are likely involved in the response to salt stress. The increased salt tolerance in the HO line might be related to the higher expression of genes that are up-regulated by salt stress, potentially enhancing the salt tolerance of HO peanuts.

Through GO analysis, we found that key genes encoding enzymes involved in lignin biosynthesis, including caffeic acid/5-hydroxyferulic acid O-methyltransferase (COMT, gene IDs: arahy.684P3R, arahy.V6JT5K), cinnamyl dehydrogenase (CAD, gene IDs: arahy.SMYQ1X, arahy.ZA4BYI), and caffeoyl CoA O-methyltransferase class proteins (CCoAOMT, gene ID: arahy.RBR1AL), were upregulated in HO and HY20 after salt treatment (Figure 7C). For example, the expression level of the key enzyme for lignin biosynthesis, AhCOMT (arahy.684P3R), is found to be twice as high in HO peanut leaves under normal conditions compared to HY20. Upon exposure to salt stress, the expression of AhCOMT genes in both HY20 and HO peanut leaves is upregulated, with an increase of 3.3 and 2.4 times, respectively, and HO leaves had higher expression levels. These expression patterns suggest that lignin biosynthesis is responsive to salt stress, consistent with the observed promotion of vessel differentiation in peanut roots under saline conditions.

## 3. Discussion

Previously, we obtained a high-oil peanut line through the use of pingyangmycin-induced mutagenesis and hydroxyproline screening [11]. This line exhibited increased tolerance to salt stress when grown in saline-alkali soil and under salt treatment in an artificial climate chamber. It suggested a potential relationship between the oil content of peanuts and their ability to salt tolerance. In this study, we used the high-oil line and its parent as plant materials to investigate the gene expression and physiological and biochemical changes in peanuts under salt stress. To elucidate the relationship between high oil content and salt tolerance, we analyzed DEGs between the high-oil line and its parent with and without the salt treatment. Our goal is to provide insights into the underlying mechanisms linking oil content with the plant’s salt tolerance.

Peanuts are moderately salt tolerant plants. Peanut leaves contain large water storage tissues, developed palisade tissues, and mechanical tissues, which are rich in chloroplasts and abundant crystal idioblasts. Crystal idioblasts, known for their ability to accumulate and sequester calcium oxalate crystals are a typical drought-resistant structure [32,33]. Recent studies have shown a close relationship between calcium oxalate crystals and plant salt-alkali resistance [34]. Under salt stress, the concentration of calcium ions in plants increases, leading to the formation of calcium oxalate crystals. These crystals help to mitigate the toxicity of excess calcium and oxalic acid to plant cells. The water storage function of cells containing these crystals also enhances plant tolerance to dehydration induced by salt stress. The root is the first plant organ to detect limitations in water supply in the soil, and a defective root system due to damage from salt stress impairs plant survival [35]. The advanced and enhanced vascular development enhanced the absorption and transportation of water and mineral nutrients, which is essential for maintaining overall plant growth despite the osmotic stress imposed by high salt levels [36]. In our study, we found that HO line relied on a well-functioning root and leaf system under salt stress, facilitating water availability for the maintenance of its above-ground tissues. As a result, HO exhibited higher levels of photosynthetic pigments, membrane stability index, osmolytes, and antioxidant activities during salt stress.

When plants are exposed to salt stress, they often produce and accumulate large amounts of reactive oxygen species (ROS), such as H_2_O_2_ and O_2_^•−^. These ROS can oxidize macromolecules like nucleic acids, proteins, and lipids, leading to oxidative damage and disruption of the plant’s metabolic balance [37,38]. POD, SOD, CAT, and other antioxidant enzymes can effectively scavenge ROS [39]. Numerous studies have shown that salt-tolerant species enhance their salt tolerance by strengthening their antioxidant defense system under salt stress [40,41]. In this study, the HO line exhibited a more robust antioxidative response compared to the HY20 line during exposure to salt stress., clearing excess ROS and thus reducing their accumulation and minimizing oxidative damage oxidation in peanut seedlings. We deduced that HO displays a more efficient “first line” of defense against ROS under salt stress conditions, thereby experiencing less severe oxidative stress. The timely removal of ROS produced under salt stress is essential for the maintenance and restoration of photosynthesis [42]. We found that when the salt concentration in the environment reached 300 mmol/L, the HO line reduced water loss and maintained a relatively high photosynthetic efficiency. This dual strategy of water conservation and photosynthetic efficiency maintenance is indicative of the HO line’s tolerance under salt stress.

Salt tolerance in plants is frequently associated with their ability to regulate the Na^+^/K^+^ ratio within cells [43]. A high level of salt tolerance is often characterized by a reduced Na^+^/K^+^ ratio, indicating that the plant has effectively managed the uptake and compartmentalization of these ions to maintain cellular homeostasis [44,45]. Our results show that the HO line exhibits a higher salt tolerance compared to the HY20 line, as evidenced by a lower Na^+^/K^+^ ratio under salt stress conditions. This suggests that the HO line has a more efficient strategy for Na^+^ exclusion and K^+^ retention. The ability to maintain a lower Na^+^/K^+^ ratio is indicative of a robust ionic homeostasis mechanism that helps protect the plant from the detrimental effects of high salinity, thereby supporting its survival and growth in saline soils. Ca^2+^ acts as a vital secondary messenger in plants, playing a crucial role in signaling pathways that respond to various environmental stimuli, including salt stress. Rapid and transient increases in cytosolic Ca^2+^ levels are known to initiate a cascade of salt tolerance responses [46,47]. We observed that under salt treatment, the Ca^2+^ content in the HO line was significantly higher than that in the HY20 line, highlighting the importance of calcium signaling in the plant’s response to saline stress.

Under unfavorable conditions such as salt stress, plants can alter the content and composition of membrane lipids to adapt to the effects of adverse factors [48,49]. Unsaturated fatty acids, particularly linolenic acid (18:3), are essential components of membrane glycerolipids and play a critical role in maintaining membrane fluidity, integrity, and function under abiotic stress conditions [50,51]. The degree of fatty acid unsaturation influences the physical properties of the lipid bilayer, with higher unsaturation generally associated with greater membrane fluidity and resilience to stress-induced phase transitions [52]. In peanut, salt stress has been shown to alter the activity of ω-3 fatty acid desaturases and the composition of unsaturated fatty acids, linking membrane lipid remodeling to stress adaptation [29]. In the present study, we observed a significant increase in linolenic acid content in the HO line under salt stress compared to HY20 (Figure 5G). This increase may contribute to the maintenance of membrane stability and functionality under saline conditions, thereby supporting the enhanced salt tolerance of the HO line. These findings are consistent with previous reports that salt-tolerant plant genotypes often exhibit higher levels of polyunsaturated fatty acids under stress, which help preserve membrane integrity and prevent ion leakage [29,53].

The transcriptomic analysis revealed distinct expression profiles between HO and HY20, with 76% of DEGs between the two lines also responding to salt stress, suggesting that HO possesses a pre-activated stress-related transcriptional landscape. Gene Ontology (GO) enrichment analysis among these overlapping genes highlighted several biologically relevant categories. Notably, terms associated with jasmonic acid (JA) and salicylic acid (SA) signaling pathways were significantly enriched, alongside regulation of hydrogen peroxide metabolic process and response to water deprivation. These findings are consistent with our physiological observations showing higher antioxidant enzyme activities (SOD, POD, CAT) and lower ROS accumulation in HO under salt stress, indicating that HO may have a primed hormonal and oxidative stress signaling network. In addition, genes related to fatty acid metabolism was enriched among DEGs between HO and HY20, which consistent with our physiological observation that HO accumulated significantly higher levels of linolenic acid under salt stress (Figure 5G). This suggests that HO may possess an enhanced capacity for membrane lipid remodeling, which is known to contribute to membrane stability and stress resilience [29].

Genes encoding caffeic acid O-methyltransferase (*AhCOMT*, arahy.684P3R) and cinnamyl alcohol dehydrogenase (*AhCAD*, arahy.SMYQ1X) were significantly upregulated in HO under both control and salt stress conditions. Lignin reinforces xylem vessels to prevent collapse under osmotic stress-induced negative pressure, thereby maintaining root hydraulic conductivity [36]. The transcriptional upregulation of these two key lignin biosynthetic genes was consistent with enhanced xylem differentiation and larger vessel diameter in HO roots (Figure 3), suggesting that enhanced lignin deposition contributes to structural reinforcement and restricted Na^+^ transport under salt stress. This may explain the lower leaf Na^+^/K^+^ ratio (Figure 5) and superior photosynthetic performance of HO under salt stress. The involvement of lignin biosynthesis in salt tolerance is further supported by studies in other species, including Arabidopsis [54], alfalfa [55], and the halophyte *Sesuvium portulacastrum* L. [56]. The higher expression of lignin biosynthesis genes in HO highlights these genes as promising targets for molecular breeding aimed at improving salt tolerance in peanuts and potentially other crops.

Taken together, our multi-dimensional data support an integrated model explaining the superior salt tolerance of the high-oil HO line relative to its parental line HY20, with synergistic regulation across transcriptional, physiological, and anatomical levels. At the transcriptional level, the HO line exhibits constitutive and salt-induced upregulation of key genes in lignin biosynthesis, ROS scavenging, ionic homeostasis, and photosystem maintenance, establishing a pre-adapted transcriptional state for salt stress response. This transcriptional reprogramming drives two core physiological adaptations: first, a robust antioxidant system that alleviates salt-induced oxidative damage, protects cell membrane integrity, and preserves the stability of lipid biosynthesis-related enzymes; second, efficient ionic homeostasis regulation that limits excess Na^+^ acropetal transport and maintains optimal K^+^/Na^+^ ratio in aerial tissues. At the anatomical level, the upregulation of lignin biosynthetic genes leads to enhanced xylem differentiation and thicker cell walls in HO roots, which not only reinforces root structural integrity to maintain hydraulic conductivity under osmotic stress, but also restricts excessive Na^+^ uptake and transport to leaves. The combined effects of these multi-level adaptations ultimately protect the photosynthetic apparatus of HO from salt-induced damage, maintaining high photosynthetic efficiency to support both vegetative growth and carbon supply for oil biosynthesis under salt stress. This model reveals a potential synergistic link between oil accumulation and salt tolerance in peanut, with multi-layered complementary adaptations collectively conferring robust salt tolerance to the high-oil line.

While this study provides comprehensive insights into the salt tolerance mechanisms of the high-oil peanut line HO, several limitations should be acknowledged. The investigation was limited to only two peanut genotypes. Although this paired comparison allowed direct assessment of the effects of mutagenesis-induced high-oil trait on salt tolerance, it does not capture the full genetic diversity of salt tolerance mechanisms across a broader range of peanut germplasm. In addition, while the correlation between gene expression patterns and physiological/anatomical observations supports their potential involvement, functional validation of the candidate genes identified in this study will be essential to establish their causal roles in salt tolerance. Despite these limitations, the present study provides a robust foundation for understanding the multi-level mechanisms linking oil accumulation to salt tolerance in peanut, and the candidate genes and traits identified offer valuable targets for future functional studies and breeding programs. Future studies will focus on the functional validation of these key candidate genes, and the in-depth dissection of the molecular regulatory network linking oil accumulation and salt tolerance in peanut, to provide more solid theoretical support for the breeding of high-oil, salt-tolerant peanut varieties.

## 4. Conclusions

In this study, we systematically compared the salt tolerance of a high-oil peanut line (HO, 58% oil content) derived from mutagenesis and its parental line HY20 (52% oil content) through anatomical, physiological, and transcriptomic analyses. Our results demonstrated that HO exhibited better salt tolerance relative to HY20, supported by better-developed root and leaf structures, higher antioxidant enzyme activities, stronger photosynthetic efficiency, and more efficient ionic homeostasis under salt stress. Transcriptomic analysis suggested that the HO line may possess a pre-activated stress-related transcriptional landscape, and upregulated lignin biosynthesis-related genes in HO may contribute to its enhanced salt tolerance. This study established a potential link between high oil content and salt tolerance in peanuts, providing theoretical support and valuable germplasm for breeding high-yield, high-oil, salt-tolerant peanut varieties suitable for saline soils.

## 5. Methods

### 5.1. Plant Materials and Growth Conditions

The high-oil line (HO) was developed from the peanut variety Huayu 20 (HY20) through mutagenesis with pingyangmycin (PYM), using hydroxyproline (HYP) as a selection factor to obtain genetically stable peanut lines with varying oil contents [11].

Peanut seeds were surface-sterilized with 5% sodium hypochlorite for 5 min, followed by rinses with sterile distilled water. The sterilized seeds were then placed on two layers of sterile filter paper in 12 cm diameter Petri dishes and moistened with distilled water. The covered petri dishes were incubated in a constant-temperature incubator at 25–27 °C for germination. Uniformly germinated seeds were selected for transplanting. For each experiment, the peanut seeds were germinated and then transplanted into 24 cm diameter plastic pots filled with clean, sterilized river sand. Seedlings were grown in an artificial climate chamber under a 16-h light/8-h dark photoperiod, with a light intensity of 600 μmol m^−2^ s^−1^, a temperature of 22 °C, and a relative humidity of 55%.

Seedlings were randomly assigned to control or salt treatment groups using a completely randomized design. Each treatment group consisted of 30 seedlings, divided into three biological replicates (10 plants per replicate). Cultivation continued until the four-leaf stage, at which point the salt treatment was initiated. NaCl solutions were prepared using 1/2 Hoagland solution as the base, at concentrations of 0, 50, 100, 200, and 300 mmol/L. The 50 and 100 mmol/L treatments simulate the moderate and severe salinity levels encountered in peanut cultivation. The 200 and 300 mmol/L treatments represent severe stress conditions, and these concentrations are commonly used in published studies on peanut salt tolerance [29,53]. This concentration gradient enables both agronomically relevant assessment and determination of differential tolerance limits between the HO and HY20 lines. All physiological, biochemical, and transcriptomic analyses were performed on three independent biological replicates, with three technical replicates per measurement where applicable.

### 5.2. Microscopic Observation

The peanut seedlings were cultured as above described. The leaves were carefully hand-sectioned and prepared for microscopic observation. The primary roots of peanut seedlings were cultured as follows: Seeds of the HY20 and HO lines were soaked and disinfected in 5% sodium hypochlorite for 5 min, then rinsed with distilled water. The cleaned seeds were then placed in a 12 cm diameter sterilized culture dish lined with two layers of filter paper. For the treatment group, 50 mL of a 50 mmol/L NaCl solution was added to the culture dish, while the control group received an equal volume of distilled water. The culture dishes were placed in an artificial climate chamber as same as the description in Section 5.1. The culture medium was refreshed daily, and three replicates were set for each treatment group. After 8 days, the top 1 cm of the root tips were manually sectioned and stained using a 5% ethanol and 40% hydrochloric acid solution, following the method described previously [57]. Temporary mounts were prepared for microscopic observation. Three replicates were set for each group. An Olympus optical microscope was used to observe and photograph the microstructures of the roots, and leaves. Characteristic images were captured to document the structures.

### 5.3. Photochemical Analysis of PSII

Chlorophyll fluorescence parameters of peanuts were measured by plant efficiency analyzer instrument (Handy PEA, Hansatech, King’s Lynn, UK) [58]

### 5.4. Determination of Photosynthetic Efficiency

Photosynthetic parameters were measured using a LI-6800 portable photosynthesis analyzer, following the method described previously [59]. Net photosynthetic rate, stomatal conductance (Gs), and water use efficiency (WUE) were determined for the third fully expanded leaves of the peanut plants.

### 5.5. Determination of the Content of the ROS, MDA and the Antioxidant Enzyme Activities

The rate of superoxide anion production, the content of hydrogen peroxide and the antioxidant enzyme activities, superoxide dismutase (SOD), catalase (CAT) and proxidase (POD) of peanuts were measured as described previously [60,61,62]. MDA contents were measured by the thiobarbituric acid (TBA) assay [63].

### 5.6. Determination of Na^+^, K^+^, and Ca^2+^ Content

The Na^+^, K^+^, and Ca^2+^ contents were determined as follows: Peanut seedling root, stem, and leaf samples were pretreated and then digested using an MDS-2002A (Shanghai Xinyi Microwave Chemistry Technology Co., Ltd., Shanghai, China) microwave digestion oven. The ion concentrations were quantified using an SP-3520A (Shanghai Spectrum Instruments Co., Ltd., Shanghai, China) atomic absorption atomic absorption spectrophotometer. Prior to analysis, standard solutions of Na^+^, K^+^, and Ca^2+^ were prepared to construct calibration curves. The ion content in the samples was calculated based on these calibration curves [64,65].

### 5.7. Determination of the Fatty Acid Content

After washing peanut seedlings subjected to salt stress with ultrapure water and collecting leaves and roots, the samples were frozen for 2 h in an ultra-low temperature refrigerator and then freeze-dried for 36 h. The dried samples were ground into a powder. Lipid extraction was performed using a mixture of methanol and chloroform [66]. After lipid extraction, FAMEs were directly obtained by transesterification using a solution of methanol/sulfuric acid [67]. Determinations of FAMEs were carried out on an Agilent Gas Chromatography/Mass Spectrometry (Agilent Technologies, Inc., Santa Clara, CA, USA).

### 5.8. Statistical Analysis

Before formal statistical comparisons, the normality of data distribution was tested using the Shapiro-Wilk test, and the homogeneity of variances was verified using Levene’s test. For datasets meeting the assumptions of normality and homogeneity of variances, Student’s *t*-test was used for pairwise comparisons between the HO and HY20 lines under the same NaCl treatment. The mean value of the 3 technical replicates was first calculated to obtain the final measurement value for each biological replicate. All formal statistical analyses were performed using the mean values of the 3 independent biological replicates as the statistical unit. Data are presented as mean ± standard error (SE) from three biological replicates, each with three technical replicates. Statistically significant differences were defined at the threshold of *p* < 0.05, and extremely significant differences were defined at *p* < 0.01.

### 5.9. Total RNA Isolation

RNA samples were from HO line and HY20 of peanut leaves treated with and without 200 mmol/L NaCl treatment, respectively, for 12 h. Total RNA was extracted using TRIZOL^®^ reagent (Invitrogen, Carlsbad, CA, USA) and purified using Qiagen RNeasy columns (Qiagen, Hilden, Germany).

### 5.10. RNA-Seq Analysis

Library and sequencing were performed on the Illumina NextSeq platform to generate 150 bp paired-end reads by the company Annoroad Gene Technology Beijing Co. Ltd., Beijing, China Each group had three replicates (Supplemental Table S1). RNA-seq data with 3 biological replicates were generated. The chromosome-level reference genome of cultivated peanut (*Arachis hypogaea* cv. Tifrunner v2.0) and the corresponding gene structure annotation file (GFF3) were downloaded from the PeanutBase database (https://peanutbase.org/, accessed on 23 August 2022). Sequencing reads of RNA-seq were aligned to the reference genome using HISAT2 (version 2.2.1) with default parameters [68]. Only uniquely mapped reads with mapping quality ≥ 20 were retained for downstream analysis. Gene expression levels were quantified, and differential expression analysis was performed using Cufflinks (version 2.2.1) [69]. Differentially expressed genes (DEGs) were identified using the following criteria: |log_2_(fold change)| ≥ 1 and false discovery rate (FDR) ≤ 0.05. GO enrichment analysis was performed using the agriGOv2 website [70] and REVIGO [71], with FDR < 0.05 considered statistically significant.

## Data Availability

The raw data supporting the conclusions of this article will be made available by the authors on request.

## Supplementary Materials

The following supporting information can be downloaded at: https://www.mdpi.com/article/10.3390/plants15081193/s1, Supplementary Figure S1. Differentially expressed genes under salt stress in peanut leaves of HO and HY20 lines. (A,B) Venn diagram of DEGs salt stress in high-oil lines and parents. A, up-regulated genes during NaCl treatment; B, down-regulated genes during NaCl treatment. (C,D) Selected enriched GO terms of up-regulated genes (C) and down-regulated genes (D) under salt stress. The scatterplot shows the enriched GO terms in a two dimensional space derived by the GO terms’ semantic similarities. Bubble color indicates the log2FDR; size indicates the frequency of the GO term in the underlying GOA database; Supplementary Figure S2. SEACOMPARE analysis for DEGs under salt treatment and peanuts lines with different oil contents. grey color represents no significant enrichment; Supplemental Figure S3. Related genes with both salt stress and oil contents. (A) Up-regulated genes related with DEGs in parents and high-oil lines. (B) Down-regulated genes related with DEGs in parents and high-oil lines; Supplementary Table S1. Statistical summary of RNA sequencing libraries; Supplementary Data S1. The differentially expressed genes in HY20 during salt stress; Supplementary Data S2. The differentially expressed genes in HO lines during salt stress; Supplementary Data S3. GO enrichment analysis for up- or down-regulated genes under salt stress; Supplementary Data S4. The DEG genes between high-oil lines and parents; Supplementary Data S5. GO enrichment analysis for DEGs between different lines; Supplementary Data S6. Genelist of the overlapped genes between high-oil related and salt-related genes; Supplementary Data S7. GO enrichment analysis for overlapped genes between high-oil related and salt-related genes.

## References

[1] Hasanuzzaman M., Raihan M.R.H., Masud A.A.C., Rahman K., Nowroz F., Rahman M., Nahar K., Fujita M. (2021). Regulation of Reactive Oxygen Species and Antioxidant Defense in Plants under Salinity. Int. J. Mol. Sci..

[2] Schieber M., Chandel Navdeep S. (2014). ROS Function in Redox Signaling and Oxidative Stress. Curr. Biol..

[3] Su L.J., Zhang J.H., Gomez H., Murugan R., Hong X., Xu D., Jiang F., Peng Z.Y. (2019). Reactive Oxygen Species-Induced Lipid Peroxidation in Apoptosis, Autophagy, and Ferroptosis. Oxidative Med. Cell. Longev..

[4] Sachdev S., Ansari S.A., Ansari M.I., Fujita M., Hasanuzzaman M. (2021). Abiotic Stress and Reactive Oxygen Species: Generation, Signaling, and Defense Mechanisms. Antioxidants.

[5] Malakar P., Chattopadhyay D. (2021). Adaptation of plants to salt stress: The role of the ion transporters. J. Plant Biochem. Biotechnol..

[6] Asif M.A., Zafar Y., Iqbal J., Iqbal M.M., Rashid U., Ali G.M., Arif A., Nazir F. (2011). Enhanced expression of AtNHX1, in transgenic groundnut (*Arachis hypogaea* L.) improves salt and drought tolerence. Mol. Biotechnol..

[7] Xu Y., Zhang Z., Ding H., Wen S., Zhang G., Qin F., Dai L. (2021). Comprehensive effects of salt stress and peanut cultivars on the rhizosphere bacterial community diversity of peanut. Arch. Microbiol..

[8] Gundaraniya S.A., Ambalam P.S., Tomar R.S. (2020). Metabolomic Profiling of Drought-Tolerant and Susceptible Peanut (*Arachis hypogaea* L.) Genotypes in Response to Drought Stress. ACS Omega.

[9] Li P., Peng Z., Xu P., Tang G., Ma C., Zhu J., Shan L., Wan S. (2021). Genome-Wide Identification of NAC Transcription Factors and Their Functional Prediction of Abiotic Stress Response in Peanut. Front. Genet..

[10] Mallikarjuna G., Rao T.S.R.B., Kirti P.B. (2016). Genetic engineering for peanut improvement: Current status and prospects. Plant Cell Tissue Organ Cult. (PCTOC).

[11] Wang J.-S., Shi L., Liu Y., Zhao M.-X., Wang X., Qiao L.-X., Sui J.-M., Li G., Zhu H., Yu S.-L. (2020). Development of peanut varieties with high oil content by in vitro mutagenesis and screening. J. Integr. Agric..

[12] Patel M., Fatnani D., Parida A.K. (2025). Mineral nutrient acquisition, antioxidative defense, and metabolomic responses in divergent genotypes of *Arachis hypogaea* L. (peanut) for salinity resilience at early seedling stage. Plant Sci..

[13] Bertioli D.J., Cannon S.B., Froenicke L., Huang G., Farmer A.D., Cannon E.K., Liu X., Gao D., Clevenger J., Dash S. (2016). The genome sequences of *Arachis duranensis* and *Arachis ipaensis*, the diploid ancestors of cultivated peanut. Nat. Genet..

[14] Bertioli D.J., Jenkins J., Clevenger J., Dudchenko O., Gao D., Seijo G., Leal-Bertioli S.C.M., Ren L., Farmer A.D., Pandey M.K. (2019). The genome sequence of segmental allotetraploid peanut Arachis hypogaea. Nat. Genet..

[15] Chen X., Li H., Pandey M.K., Yang Q., Wang X., Garg V., Li H., Chi X., Doddamani D., Hong Y. (2016). Draft genome of the peanut A-genome progenitor (*Arachis duranensis*) provides insights into geocarpy, oil biosynthesis, and allergens. Proc. Natl. Acad. Sci. USA.

[16] Xue H., Zhao K., Zhao K., Han S., Chitikineni A., Zhang L., Qiu D., Ren R., Gong F., Li Z. (2024). A near complete genome of Arachis monticola, an allotetraploid wild peanut. Plant Biotechnol. J..

[17] Yin D., Ji C., Ma X., Li H., Zhang W., Li S., Liu F., Zhao K., Li F., Li K. (2018). Genome of an allotetraploid wild peanut Arachis monticola: A de novo assembly. Gigascience.

[18] Zhuang W., Chen H., Yang M., Wang J., Pandey M.K., Zhang C., Chang W.C., Zhang L., Zhang X., Tang R. (2019). The genome of cultivated peanut provides insight into legume karyotypes, polyploid evolution and crop domestication. Nat. Genet..

[19] Han Y., Li R., Liu Y., Fan S., Wan S., Zhang X., Li G. (2021). The Major Intrinsic Protein Family and Their Function Under Salt-Stress in Peanut. Front. Genet..

[20] Dong X., Gao Y., Bao X., Wang R., Ma X., Zhang H., Liu Y., Jin L., Lin G. (2023). Multi-Omics Revealed Peanut Root Metabolism Regulated by Exogenous Calcium under Salt Stress. Plants.

[21] Yang S., Zhao L., Yan J., Zhang J., Guo F., Geng Y., Wang Q., Yang F., Wan S., Li X. (2019). Peanut genes encoding tetrapyrrole biosynthetic enzymes, AhHEMA1 and AhFC1, alleviating the salt stress in transgenic tobacco. Plant Physiol. Biochem..

[22] Wang Z., Yan L., Wan L., Huai D., Kang Y., Shi L., Jiang H., Lei Y., Liao B. (2019). Genome-wide systematic characterization of bZIP transcription factors and their expression profiles during seed development and in response to salt stress in peanut. BMC Genom..

[23] Yuan C., Li C., Lu X., Zhao X., Yan C., Wang J., Sun Q., Shan S. (2020). Comprehensive genomic characterization of NAC transcription factor family and their response to salt and drought stress in peanut. BMC Plant Biol..

[24] Luo L., Wan Q., Zhang K., Zhang X., Guo R., Wang C., Zheng C., Liu F., Ding Z., Wan Y. (2021). AhABI4s Negatively Regulate Salt-Stress Response in Peanut. Front. Plant Sci..

[25] Wang Q., Zhang Z., Guo C., Zhao X., Li Z., Mou Y., Sun Q., Wang J., Yuan C., Li C. (2023). Hsf transcription factor gene family in peanut (*Arachis hypogaea* L.): Genome-wide characterization and expression analysis under drought and salt stresses. Front. Plant Sci..

[26] Zhu H., Jiang Y., Guo Y., Huang J., Zhou M., Tang Y., Sui J., Wang J., Qiao L. (2021). A novel salt inducible WRKY transcription factor gene, AhWRKY75, confers salt tolerance in transgenic peanut. Plant Physiol. Biochem..

[27] Zhao X., Wang Q., Yan C., Sun Q., Wang J., Li C., Yuan C., Mou Y., Shan S. (2024). The bHLH transcription factor AhbHLH121 improves salt tolerance in peanut. Int. J. Biol. Macromol..

[28] Sui J., Jiang D., Zhang D., Song X., Wang J., Zhao M., Qiao L. (2016). The Salinity Responsive Mechanism of a Hydroxyproline-Tolerant Mutant of Peanut Based on Digital Gene Expression Profiling Analysis. PLoS ONE.

[29] Sui N., Wang Y., Liu S., Yang Z., Wang F., Wan S. (2018). Transcriptomic and Physiological Evidence for the Relationship between Unsaturated Fatty Acid and Salt Stress in Peanut. Front. Plant Sci..

[30] Gupta K., Kayam G., Faigenboim-Doron A., Clevenger J., Ozias-Akins P., Hovav R. (2016). Gene expression profiling during seed-filling process in peanut with emphasis on oil biosynthesis networks. Plant Sci..

[31] Yin D., Wang Y., Zhang X., Li H., Lu X., Zhang J., Zhang W., Chen S. (2013). De novo assembly of the peanut (*Arachis hypogaea* L.) seed transcriptome revealed candidate unigenes for oil accumulation pathways. PLoS ONE.

[32] Li Z., Li R. (1981). Anatomical observation on assimilating branches of nine xerophytes in Gansu Province, China. Acta Bot. Sin..

[33] Nakata P.A., Kostman T.A., Franceschi V.R. (2003). Calreticulin is enriched in the crystal idioblasts of *Pistia stratiotes*. Plant Physiol. Biochem..

[34] Fu Y., Lü Y., Liang X., Zou X., Yang Y., Liang S., Xun E., Wang G. (2021). Research Progress of Plant Calcium Oxalate Crystals. Mol. Plant Breed..

[35] Opitz N., Marcon C., Paschold A., Malik W.A., Lithio A., Brandt R., Piepho H.P., Nettleton D., Hochholdinger F. (2016). Extensive tissue-specific transcriptomic plasticity in maize primary roots upon water deficit. J. Exp. Bot..

[36] Agustí J., Blázquez M.A. (2020). Plant vascular development: Mechanisms and environmental regulation. Cell. Mol. Life Sci..

[37] Miller G., Suzuki N., Ciftci-Yilmaz S., Mittler R. (2010). Reactive oxygen species homeostasis and signalling during drought and salinity stresses. Plant Cell Environ..

[38] Mittler R., Vanderauwera S., Gollery M., Van Breusegem F. (2004). Reactive oxygen gene network of plants. Trends Plant Sci..

[39] Gill S.S., Tuteja N. (2010). Reactive oxygen species and antioxidant machinery in abiotic stress tolerance in crop plants. Plant Physiol. Biochem..

[40] Challabathula D., Analin B., Mohanan A., Bakka K. (2022). Differential modulation of photosynthesis, ROS and antioxidant enzyme activities in stress-sensitive and -tolerant rice cultivars during salinity and drought upon restriction of COX and AOX pathways of mitochondrial oxidative electron transport. J. Plant Physiol..

[41] Xiao F., Zhou H. (2022). Plant salt response: Perception, signaling, and tolerance. Front. Plant Sci..

[42] Li J., Liu Y., Zhang M., Xu H., Ning K., Wang B., Chen M. (2022). Melatonin increases growth and salt tolerance of Limonium bicolor by improving photosynthetic and antioxidant capacity. BMC Plant Biol..

[43] Munns R., Tester M. (2008). Mechanisms of salinity tolerance. Annu. Rev. Plant Biol..

[44] Orsini F., D’Urzo M.P., Inan G., Serra S., Oh D.H., Mickelbart M.V., Consiglio F., Li X., Jeong J.C., Yun D.J. (2010). A comparative study of salt tolerance parameters in 11 wild relatives of Arabidopsis thaliana. J. Exp. Bot..

[45] Wu H.J., Zhang Z., Wang J.Y., Oh D.H., Dassanayake M., Liu B., Huang Q., Sun H.X., Xia R., Wu Y. (2012). Insights into salt tolerance from the genome of Thellungiella salsuginea. Proc. Natl. Acad. Sci. USA.

[46] Knight H., Trewavas A.J., Knight M.R. (1997). Calcium signalling in Arabidopsis thaliana responding to drought and salinity. Plant J..

[47] Knight M.R., Campbell A.K., Smith S.M., Trewavas A.J. (1991). Transgenic plant aequorin reports the effects of touch and cold-shock and elicitors on cytoplasmic calcium. Nature.

[48] Li N., Shao T., Xu L., Long X., Rengel Z., Zhang Y. (2024). Transcriptome analysis reveals the molecular mechanisms underlying the enhancement of salt-tolerance in Melia azedarach under salinity stress. Sci. Rep..

[49] Sun M., Liu X., Gao H., Zhang B., Peng F., Xiao Y. (2022). Phosphatidylcholine Enhances Homeostasis in Peach Seedling Cell Membrane and Increases Its Salt Stress Tolerance by Phosphatidic Acid. Int. J. Mol. Sci..

[50] Mikami K., Murata N. (2003). Membrane fluidity and the perception of environmental signals in cyanobacteria and plants. Prog. Lipid Res..

[51] Upchurch R.G. (2008). Fatty acid unsaturation, mobilization, and regulation in the response of plants to stress. Biotechnol. Lett..

[52] Los D.A., Murata N. (2004). Membrane fluidity and its roles in the perception of environmental signals. Biochim. Biophys. Acta (BBA)—Biomembr..

[53] Zhang N., Zhang H., Lv Z., Bai B., Ren J., Shi X., Kang S., Zhao X., Yu H., Zhao T. (2024). Integrative multi-omics analysis reveals the crucial biological pathways involved in the adaptive response to NaCl stress in peanut seedlings. Physiol. Plant..

[54] Chun H.J., Baek D., Cho H.M., Lee S.H., Jin B.J., Yun D.J., Hong Y.S., Kim M.C. (2019). Lignin biosynthesis genes play critical roles in the adaptation of Arabidopsis plants to high-salt stress. Plant Signal Behav..

[55] Rahman M.A., Woo J.H., Lee S.H., Park H.S., Kabir A.H., Raza A., El Sabagh A., Lee K.W. (2022). Regulation of Na^+^/H^+^ exchangers, Na^+^/K^+^ transporters, and lignin biosynthesis genes, along with lignin accumulation, sodium extrusion, and antioxidant defense, confers salt tolerance in alfalfa. Front. Plant Sci..

[56] Li Y., Zhang T., Kang Y., Wang P., Yu W., Wang J., Li W., Jiang X., Zhou Y. (2023). Integrated metabolome, transcriptome analysis, and multi-flux full-length sequencing offer novel insights into the function of lignin biosynthesis as a *Sesuvium portulacastrum* response to salt stress. Int. J. Biol. Macromol..

[57] Zhu D., Tang D., Li M., Liu X. (2010). Comparison of Three Sectioning Methods for Observing the Xylem of Plants. Hunan Agric. Sci..

[58] van Kooten O., Snel J.F. (1990). The use of chlorophyll fluorescence nomenclature in plant stress physiology. Photosynth. Res..

[59] Ran X., Wang X., Gao X., Liang H., Liu B., Huang X. (2021). Effects of salt stress on the photosynthetic physiology and mineral ion absorption and distribution in white willow (*Salix alba* L.). PLoS ONE.

[60] Radwan D.E.M., Mohamed A.K., Fayez K.A., Abdelrahman A.M. (2019). Oxidative stress caused by Basagran^®^. herbicide is altered by salicylic acid treatments in peanut plants. Heliyon.

[61] Velikova V., Yordanov I., Edreva A. (2000). Oxidative stress and some antioxidant systems in acid rain-treated bean plants: Protective role of exogenous polyamines. Plant Sci..

[62] Willekens H., Chamnongpol S., Davey M., Schraudner M., Langebartels C., Van Montagu M., Inze D., Van Camp W. (1997). Catalase is a sink for H_2_O_2_ and is indispensable for stress defence in C3 plants. EMBO J..

[63] Zhang L., Tian L.H., Zhao J.F., Song Y., Zhang C.J., Guo Y. (2009). Identification of an apoplastic protein involved in the initial phase of salt stress response in rice root by two-dimensional electrophoresis. Plant Physiol..

[64] Oliveira S.R., Gomes Neto J.A., Nóbrega J.A., Jones B.T. (2010). Determination of macro- and micronutrients in plant leaves by high-resolution continuum source flame atomic absorption spectrometry combining instrumental and sample preparation strategies. Spectrochim. Acta Part B At. Spectrosc..

[65] Ye G. (2018). Determination of contents of Ca, Mg, Cu and Zn in peanut by microwave digestion-atomic absorption spectrometry. China Oils Fats.

[66] Folch J., Lees M., Sloane Stanley G.H. (1957). A simple method for the isolation and purification of total lipides from animal tissues. J. Biol. Chem..

[67] Argemi-Armengol I., Villalba D., Tor M., Perez-Santaescolastica C., Purrinos L., Manuel Lorenzo J., Alvarez-Rodriguez J. (2019). The extent to which genetics and lean grade affect fatty acid profiles and volatile compounds in organic pork. PeerJ.

[68] Kim D., Langmead B., Salzberg S.L. (2015). HISAT: A fast spliced aligner with low memory requirements. Nat. Methods.

[69] Ghosh S., Chan C.K. (2016). Analysis of RNA-Seq Data Using TopHat and Cufflinks. Methods Mol. Biol..

[70] Tian T., Liu Y., Yan H., You Q., Yi X., Du Z., Xu W., Su Z. (2017). agriGO v2.0: A GO analysis toolkit for the agricultural community, 2017 update. Nucleic Acids Res.

[71] Supek F., Bosnjak M., Skunca N., Smuc T. (2011). REVIGO summarizes and visualizes long lists of gene ontology terms. PLoS ONE.

