# Supplementary material for: Anatomical, Physiological and Transcriptomic Insights into Salt Tolerance in Two Peanut Lines with Different Oil Contents
Source: Plants (Basel). 2026 Apr 13;15(8):1193. doi: 10.3390/plants15081193 (PMC13119500; doi:10.3390/plants15081193)
Supplement: Supplementary file 1 [file plants-15-01193-s001.zip › Supplemental_figs_tbls.pdf]

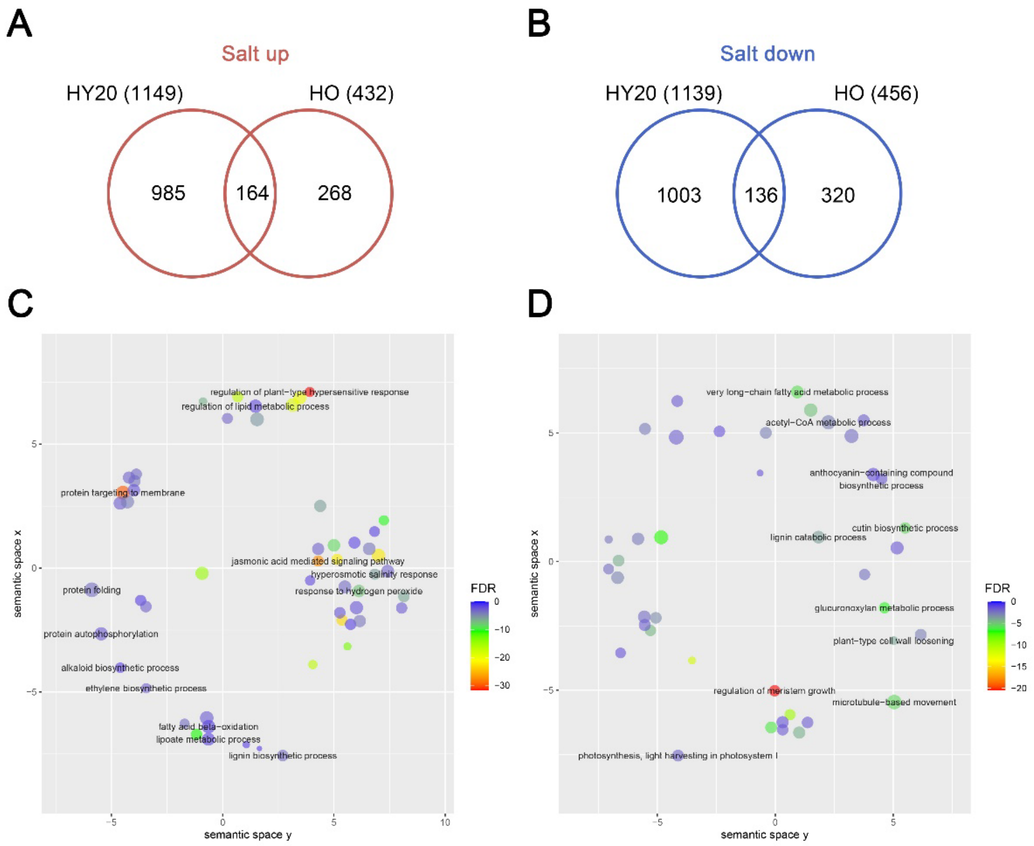

Supplementary Figure S1. Differentially expressed genes under salt stress in peanut leaves of HO and HY20 lines. (A,B) Venn diagram of DEGs salt stress in high-oil lines and parents. A, up-regulated genes during NaCl treatment; B, down-regulated genes during NaCl treatment. (C,D) Selected enriched GO terms of up-regulated genes (C) and down-regulated genes (D) under salt stress. The scatterplot shows the enriched GO terms in a two dimensional space derived by the GO terms' semantic similarities. Bubble color indicates the $\log_2$FDR; size indicates the frequency of the GO term in the underlying GOA database.

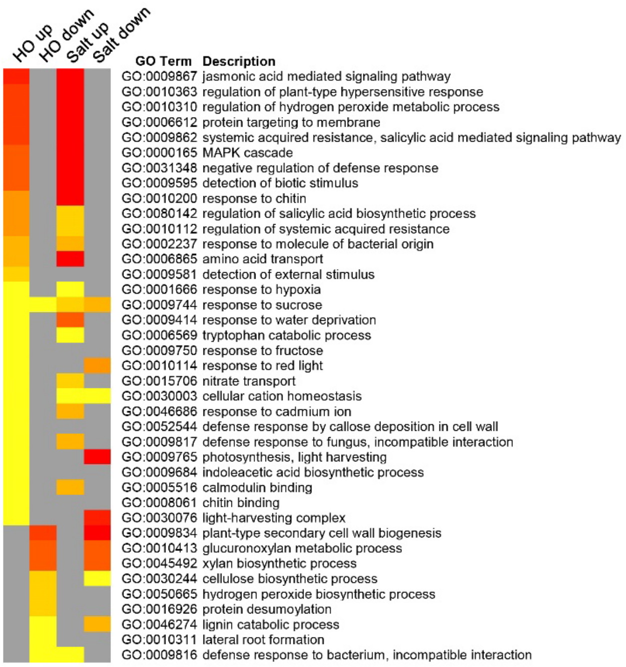

Supplementary Figure S2. SEACOMPARE analysis for DEGs under salt treatment and peanuts lines with different oil contents. grey color represents no significant enrichment.

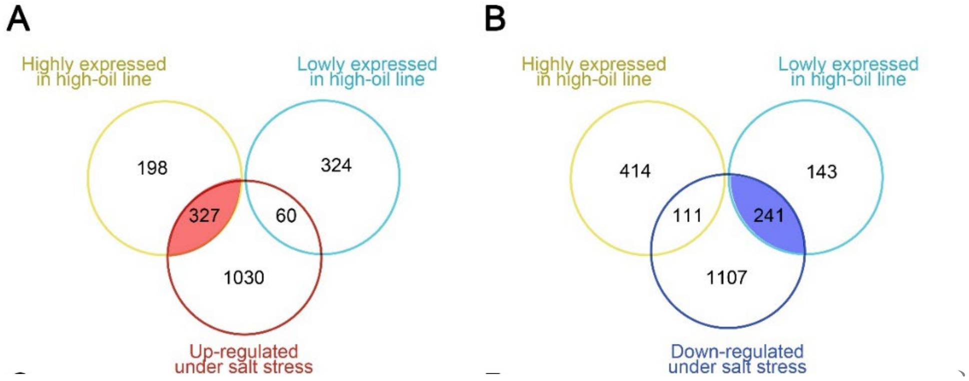

Supplementary Figure S3. Related genes with both salt stress and oil contents. (A) Up-regulated genes related with DEGs in parents and high-oil lines. (B) Down-regulated genes related with DEGs in parents and high-oil lines.

Supplementary Table S1. Statistical summary of RNA sequencing libraries

Sample	Treatment	Biological replicate	Total pairs	Aligned pairs	overall map%	concordant map%
High-oil line (HO)	NaCl	1	44897546	40811481	94.1	89.1
		2	39369187	34819769	91.7	86.4
		3	33041850	29090364	91.4	86
	Control	1	42309625	38634883	94.3	89.3
		2	38857735	34698354	92.8	87.5
		3	38029474	34126356	93.1	87.8
		3	38925908	34635933	92.5	87.1
Parent (HY20)	NaCl	1	43514951	39608067	94.2	89.2
		2	38779521	35076845	93.7	88.4

	3	36513202	32842345	93.3	87.9
	1	43086272	39114954	94	89.1
Control	2	38015897	34050134	93	87.7
	3	37607150	33497976	92.3	87.2
